# Cancer Registration in the Middle East, North Africa, and Turkey: Scope and Challenges

**DOI:** 10.1200/GO.21.00065

**Published:** 2021-07-08

**Authors:** Zahi Abdul-Sater, Ali Shamseddine, Ali Taher, Fouad Fouad, Ghassan Abu-Sitta, Ibtihal Fadhil, Raya Saab, Richard Sullivan, Salim M. Adib, Shadi Saleh, Deborah Mukherji

**Affiliations:** ^1^ Global Health Institute, American University of Beirut, Beirut, Lebanon; ^2^ Department of Hematology/Oncology, American University of Beirut Medical Center, Beirut, Lebanon; ^3^ Faculty of Health Sciences, American University of Beirut, Lebanon; ^4^ Plastic Surgery and Reconstructive Surgery, American University of Beirut Medical Center, Beirut, Lebanon, Kuwait City, Kuwait; ^5^ Eastern Mediterranean NCD Alliance; ^6^ Department of Pediatrics and Adolescent Medicine, American University of Beirut Medical Center, Beirut, Lebanon; ^7^ Institute of Cancer Policy & Conflict & Health Research Group, King's College London, London, United Kingdom

## Abstract

National cancer control strategies have been identified as essential tools for reducing and managing the growing burden of cancer in low- and middle-income countries. Cancer registration is an instrumental component of any cancer control strategy, providing the data to inform effective cancer policy. In the Middle East, North Africa, and Turkey (MENAT) region, cancer registration varies immensely and faces multifaceted challenges including protracted conflict. This study investigates and maps out the present capacities and outputs of cancer registration in the MENAT region and identifies thematic barriers facing implementation and utilization of cancer registry data.

**MATERIALS AND METHODS:**

We used a self-administered online survey with open and close-ended questions targeting national and institutional cancer registry managers in the MENAT countries.

**RESULTS:**

Registry managers from 19 MENAT countries reported the presence of 97 population-based, 48 hospital-based, and 24 pathology-based registries. Most population-based registries were well- or partially developed. Lack of accurate death records, complete medical records, and communication between stakeholders and deficiencies in trained personnel were critical challenges that were more severe in active conflict zones and neighboring conflict-affected regions. Cancer registration challenges included weak health infrastructure, absence of legislation mandating cancer registration, and disruption of cancer registration because of active conflict and loss of funding. Refugee host countries, such as Lebanon, Turkey, and Jordan, also reported conflict-related challenges including refugee mobility and lack of accurate data on forced migrants.

**CONCLUSION:**

This study provides a much-needed understanding of the current landscape and contextual challenges affecting cancer registration in the MENAT. These data are important for identifying areas on which to focus regional capacity-strengthening initiatives.

## INTRODUCTION

The global burden of cancer is disproportionately greater in low- and middle-income countries (LMICs), including countries in the Middle East, North Africa, and Turkey (MENAT) region. In the coming decades, a 60% increase in cancer burden is projected across LMICs,^1^ with the highest relative global increase in the Arab World because of multiple factors including population aging, changes in cancer risk exposure, and improved cancer diagnostics.^2^ The development of evidence-based National Cancer Control Plans (NCCPs) has been recognized as an essential policy intervention to cope with current and future cancer burden. Cancer registration, defined as the systematic collection and analysis of tumor data, is an integral foundation of any operationally successful NCCP.^3^ Cancer registration can take multiple forms including pathology, hospital, and population-based cancer registry (PBCR). PBCR has evolved to become the global and gold standard activity in cancer surveillance systems.^4^

**CONTEXT**

**Key Objective**
What is the current status of cancer registration in the Middle East, North Africa, and Turkey (MENAT)?
**Knowledge Generated**
Overview of cancer registration in the MENAT with identification of specific challenges including policy and funding deficiencies, lack of accurate death records, lack of accurate data on migrants, and conflict-related disruption is presented.
**Relevance**
Regional capacity-strengthening initiatives can be focused on the areas identified as requiring additional support to improve cancer registration and cancer control.

**FIG A1:**
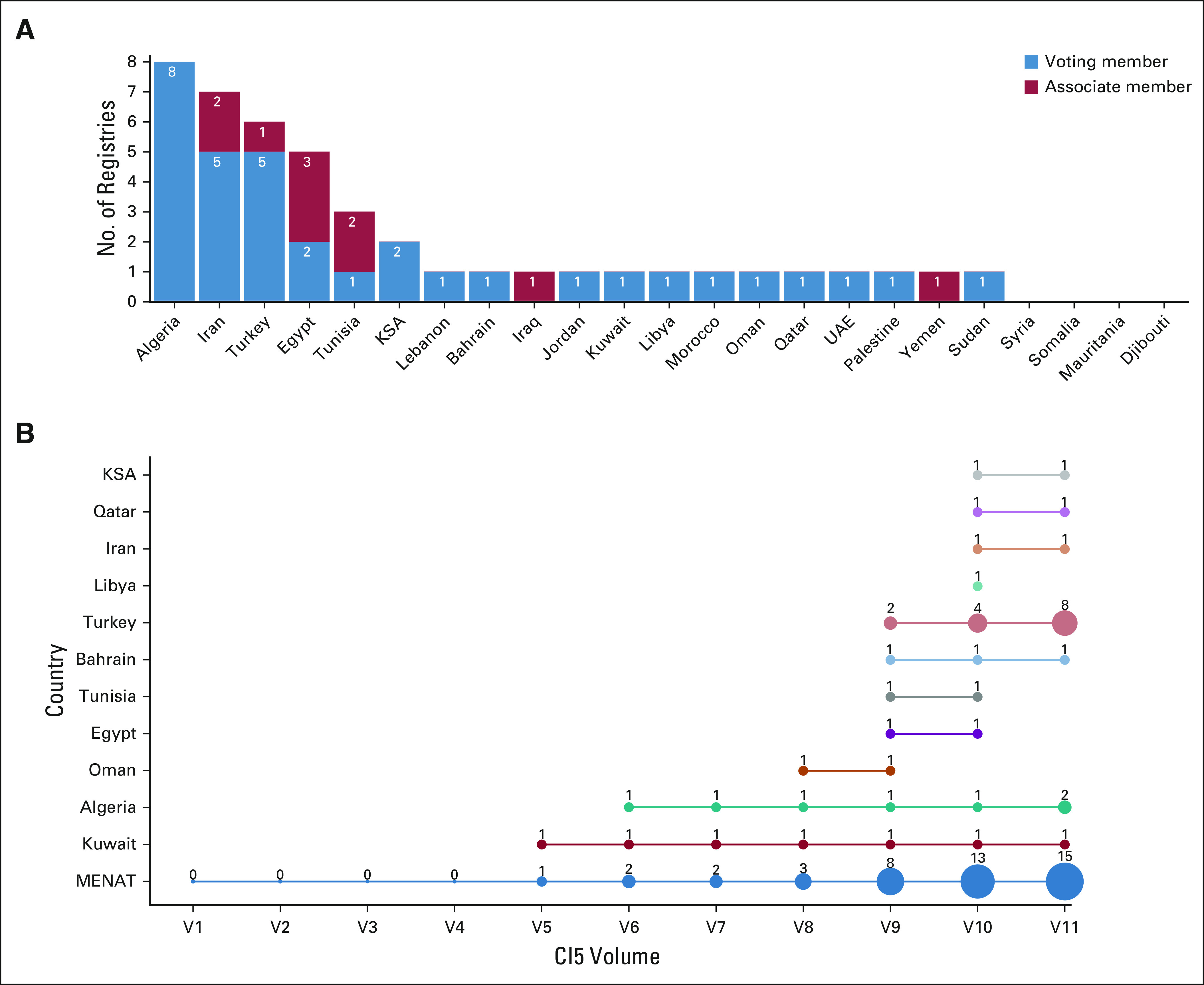
(A) MENAT PBCR that are voting or associate members in the IACR. Data extracted from IACR website on May 15, 2020. (B) Cancer registries in the MENAT region that are published in different volumes of CI5. Circle size corresponds to the number of registries published in the CI5 volume. CI5, Cancer Incidence in Five Continents; IACR, International Association of Cancer Registries; KSA, Kingdom of Saudi Arabia; MENAT, Middle East, North Africa, and Turkey; PBCR, population-based cancer registry; UAE, United Arab Emirates.

Cancer registration faces multiple obstacles in LMICs, including low resource allocation, inadequate health informatics infrastructure, lack of death records, inaccurate data, cultural taboos, and conflict-induced population mobility and instability.^5^ Importantly, according to the International Agency for Research on Cancer (IARC), PBCRs in LMICs have been developing at a much slower pace than those in high-income countries because of underinvestment and subsequent lack of human resources, despite considerable awareness on their importance.^6^ This slow growth is mirrored in the MENAT region, in terms of quantity and quality of PBCRs. Currently, 44 MENAT PBCRs are voting or associate members at the International Association of Cancer Registries (IACR). Some countries, for example, Syria, Somalia, Mauritania, and Djibouti, are not IACR members (Appendix Fig A1A). The first MENAT-based high-quality PBCR was established in Setif, Algeria. It published incidence data from 1988 to 1992 in the Cancer Incidence in Five Continents volume VI, a definitive source of high-quality incidence data of cancer.^7^ Many MENAT registries, however, do not publish in Cancer Incidence in Five Continents, either because of lack of submission or poor quality of data (Appendix Fig A1B).

The limited capacity for cancer registration in the region is due to context-specific political, social, and economic obstacles. The MENAT region has also been disproportionally affected by armed conflicts, which halted cancer registration in Syria, Yemen, and Libya. In other conflict-affected settings, the production of quality data for cancer care has been affected by an influx of refugees in Jordan and Lebanon.^8^ In Palestine, a chronic state of conflict, instability, and political and physical isolation coupled with movement restrictions makes effective reporting of cancer cases a major challenge.^9^ More recently, the MENAT region and the whole world have been profoundly affected by the novel coronavirus SARS-CoV-2, which was declared a pandemic by the WHO in March 2020.^10^ IARC has joined the global COVID-19 and Cancer Taskforce, aimed at synthesizing and disseminating data on COVID-19 and cancer.^11^ Under the umbrella of IARC, Global Initiative for Cancer Registry (GICR) and IACR sent out a survey to population-based cancer registries to assess the impact of the pandemic on cancer registration. Registries from MENAT countries, except for Saudi Arabia, reported that the pandemic has affected their registries operation and attendance of registry staff.^12^

Efforts have been made to increase the capacity of cancer registration in the MENAT region, including initiatives such as the Middle East Cancer Consortium in 1996^8^ and the Gulf Centre for Cancer Registration in 1998.^13^ In recent years, under the umbrella of the WHO, the GICR development has been providing support for cancer registration through its regional hub, based in Turkey. GICR activities include site visits, technical support, courses, and assessment workshops.^8^

Context-specific capacity-strengthening initiatives for cancer registration in the MENAT are needed, especially in the context of COVID-19 and ongoing conflicts in the region. Thus, it is essential to understand the development and operation of cancer registration in various stages of conflict in the MENAT region. It is also important to understand the nature of the contextual challenges, following a country-by-country approach, which have led to disparities in cancer registration capacities in the MENAT region. With these aims in mind, we conducted an online survey to systematically assess the landscape and challenges of cancer registration in the MENAT region.

## MATERIALS AND METHODS

### Survey Development

This study uses a self-administered online survey, in both English and Arabic Languages, composed of 25 questions related to cancer registration status in the respective country (10 questions) and cancer registry–specific questions (15 questions). The questions were adapted from a questionnaire administered during WHO/Regional Office for the Eastern Mediterranean regional meeting on cancer control and research priorities in the Eastern Mediterranean Region in 2013 convened in Doha, Qatar, in addition to published cancer registration articles and questionnaires.^14,15^

### Administration

After institutional review board approval, we used the web-based tool, LimeSurvey, to collect survey data from the cancer registry managers or administrators in the MENAT region (Table 1) who were invited to a cancer registration workshop hosted by the Global Health Institute at the American University of Beirut. An e-mail was sent directly to 26 registry managers and administrators from 19 countries in the MENAT region. The e-mail explained that the survey aimed to understand the status of national and subnational cancer registries in the MENAT. Reminder e-mails were sent 2 and 4 weeks later. The surveys were filled out between September 18, 2019, and October 11, 2019.

**TABLE 1:**
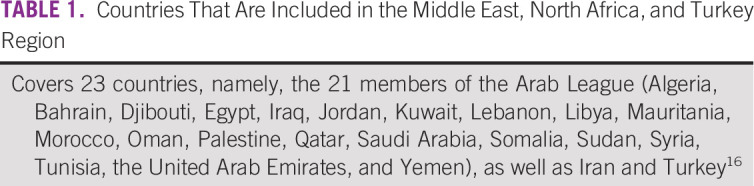
Countries That Are Included in the Middle East, North Africa, and Turkey Region

This work was granted ethical approval by the American University of Beirut Institutional Review Board, ID: SBS-2018-0492.

### Data Management

Responses were exported into Microsoft Excel, where the data were managed and cleaned, removing any incomplete entries. Figures were prepared using Microsoft Excel, Adobe Photoshop, and Flourish Studio.

## RESULTS

**FIG 1:**
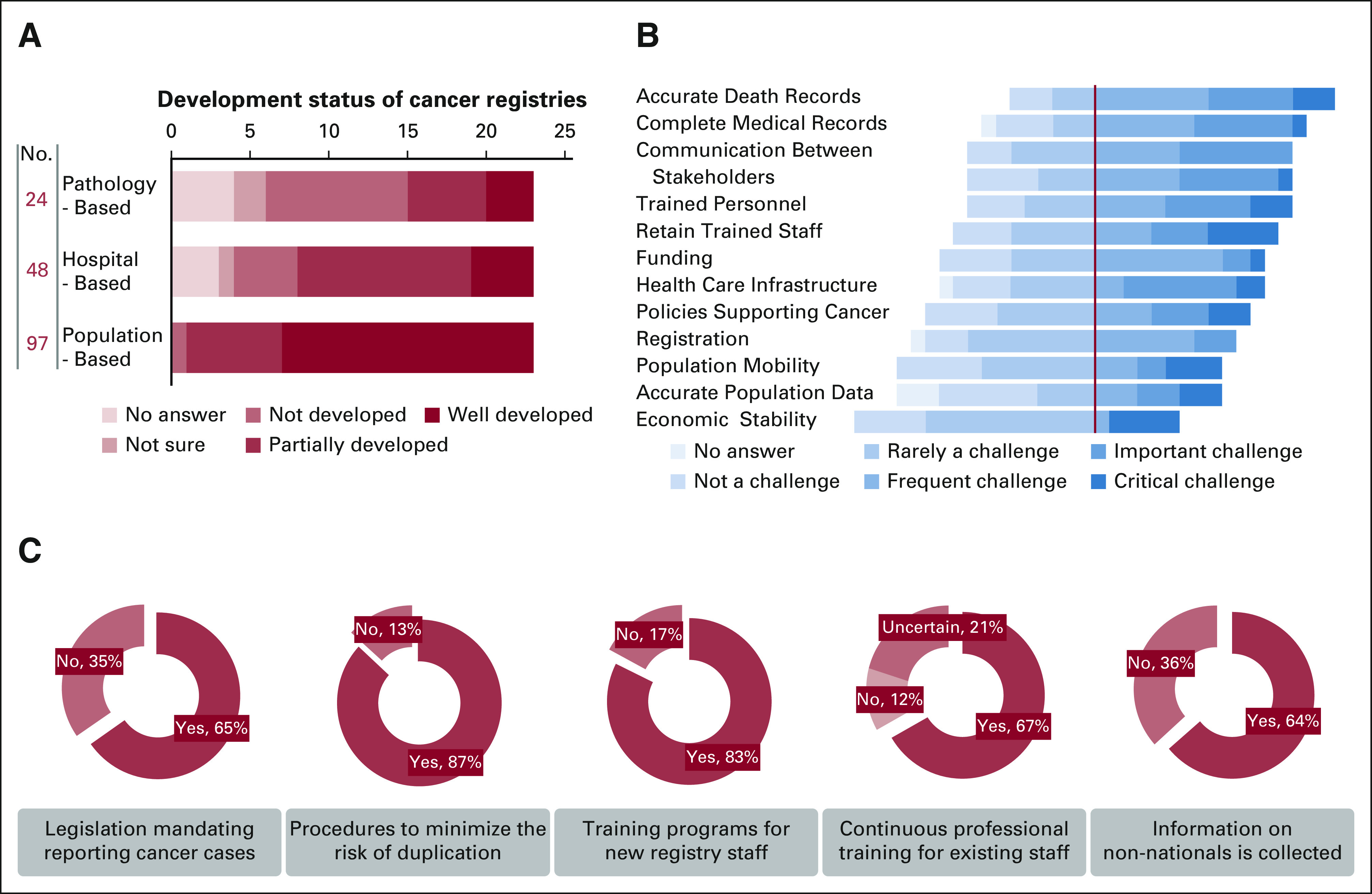
Scope and challenges of cancer registration in the MENAT. (A) Status of cancer registries in the MENAT. (B) Cancer registration challenges in the MENAT. (C) Properties of cancer registries in the MENAT. MENAT, Middle East, North Africa, and Turkey.

Data on 23 registries were received from 19 MENAT countries: Algeria, Bahrain, Egypt, Iraq, Jordan, Kuwait, Lebanon, Libya, Morocco, Oman, Palestine, Qatar, Saudi Arabia, Sudan, Tunisia, Turkey, United Arab Emirates, and Yemen (Data Supplement). Respondents reported managing 97 population-based, 48 hospital-based, and 24 pathology-based registries. Generally, respondents noted that population-based cancer registries in the MENAT are well-developed (16 of 23), hospital-based registries are partially or well-developed (15 of 20), and pathology-based registries are not developed (9 of 19) (Fig 1A). Lack of accurate death records, complete medical records, communication between stakeholders, and trained personnel was the most important and critical challenge (Fig 1B).

Most responding registries reported that there were national legislations mandating reporting cancer cases (65%), procedures to minimize the risk of duplication (87%), training programs for new registry staff (83%), continuous professional training for existing staff (67%), and collection of information on non-nationals (64%) (Fig 1C). Responding cancer registries from Algeria, Tunisia, Syria, Morocco, Palestine, and Turkey reported that they do not collect data on non-nationals. On the other hand, data are collected on expatriate work force and visitors in Qatar national cancer registry; expatriates and visitors in National Center for Cancer Care & Research registry in Qatar; international patients with cancer (mostly from neighboring Arab) in King Hussein Cancer Center Registry in Jordan; non-Jordanians including displaced populations in Jordan cancer registry; non-nationals (Refugees and Foreign Nationals) in Iraq cancer registry; Somali refugees in Aden cancer registry in Yemen; non-national patients with cancer in Oman national cancer registry; patient nationality in Benghazi cancer registry in Libya; expatriates in Kuwait; all non-nationals in Bahrain cancer registry; visitors, pilgrims, refugee, and stateless in Saudi cancer registry in Saudi Arabia; migrants and refugees from neighboring African countries and displaced populations from remote areas in Sudan national cancer registry in Sudan; and Palestinian and Syrian populations in national cancer registry in Lebanon. Responding registries from UAE and Egypt did not answer this question.

**FIG 2:**
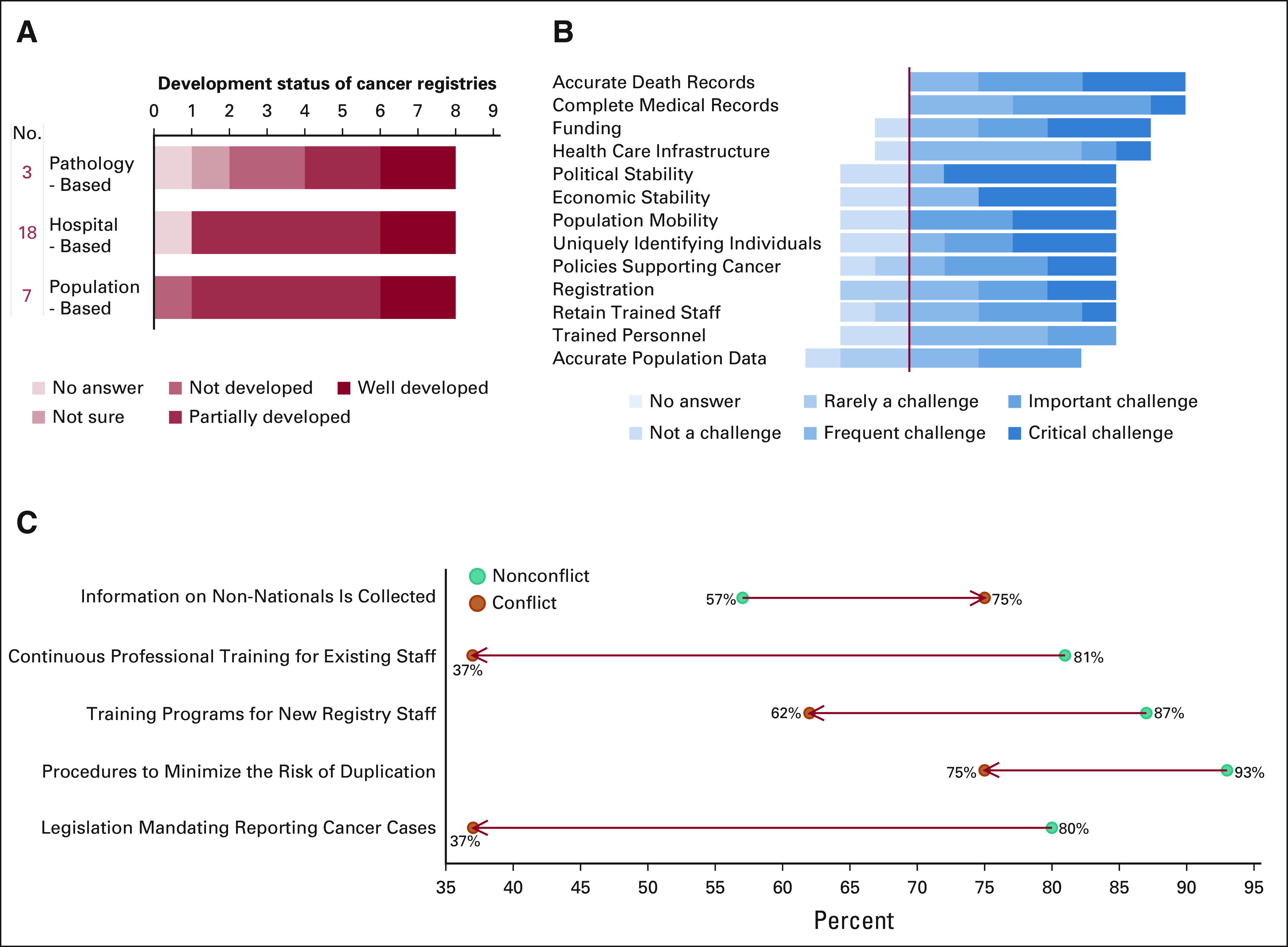
Cancer registration in conflict-affected countries in the MENAT region. (A) Status of cancer registries in conflict. (B) Cancer registration challenges in the MENAT. (C) Properties of cancer registries in the MENAT. MENAT, Middle East, North Africa, and Turkey.

Given the heterogeneity of the determinants of cancer registration capacity in the MENAT, it is important to understand the scope and challenges within conflict-affected MENAT countries. Using the World bank classification of fragile and conflict situations, conflict-affected countries in the MENAT include Djibouti, Iraq, Lebanon, Libya, Somalia, Sudan, Syria, Yemen, and Palestine; Somalia and Djibouti were not included in the study because of inability to identify or contact relevant cancer registry managers. Most registries reported in conflict-affected MENAT countries were hospital-based (18 of 28), followed by population-based (7 of 28) and pathology-based (3 of 28) (Fig 2A). Most registries based in conflict areas were partially developed, which highlights the importance of understanding the distinct challenges in these countries. In conflict-affected countries, 79% of challenges were rated frequent, important, or critical (Fig 2B) compared with 37% in nonconflict countries and 52% in all MENAT countries. Leading the challenges was also a lack of accurate death records and complete medical records. However, lack of funding, weak health care infrastructure, and political stability were also frequently reported. In comparison with nonconflict countries, conflict-affected countries reported less continuous professional training (37%), training programs (62%), risk duplication minimizing procedures (75%), and legislation mandating cancer cases reporting (37%) (Fig 2C). Interestingly, more registries reported data collection on non-nationals in conflict-affected settings. As anticipated, these data highlight that conflict-affected MENAT countries face more critical and unique challenges compared with nonconflict-affected regions.

Next, we asked survey participants to tabulate a timeline of challenges, including conflict-induced challenges. Nine registries reported that conflict-related events have affected the process of cancer registration. In Jordan, refugee influx since 2012 has caused a huge spike in non-Jordanian cancer registration figures, and in Kuwait, the Gulf War in 1990-1992 has also affected cancer registration in the country. In Turkey, refugee population mobility within the country resulted in the absence of accurate information on refugees. All conflict-affected countries except Palestine reported that cancer registration was negatively affected by conflict-related events. In Syria, cancer registration was affected by internal displacement from various cities including Rif Dimashq, Daraa, Aleppo, Quneitra, and Idlib to As-Suwayda city. In Iraq, cancer registration ceased because of armed conflicts in 2014 and the number of cancer cases is underestimated because of refugee influx since 2011. In Lebanon, population influx because of the Syrian crisis has led to modification of cancer registration numerator and denominator. In Sudan, refugee influx, including patients with cancer, from neighboring countries including Eritrea and Ethiopia affected cancer registration. Finally, military clashes all over Yemen resulted in inefficient and destroyed health facilities and cross-border travel for treatment, which entailed lack of access to medical records, loss of funding for cancer registries, and high staff turnover. The relocation of three cancer centers and the death of a tumor registrar because of the war in Yemen were also reported. Finally, in Libya, cancer registration lost its priority in the health plan during the conflict, from 2011 to 2019, and registration was affected by population migration within and outside the coverage area.

Understanding that the scope and challenges of cancer registration is contextual and specific to each country, we produced a cancer registration profile for each of the 23 registries (Data Supplement). Each cancer registry profile is composed of (1) the status of cancer registration, which includes the type, number, and status of cancer registries in the country; (2) the registry profile, which includes the number of full-time staff, the registration software, the data collection method, the data sources used (paper or electronic), and the administrative properties of the registry (legislation, duplication procedures, collected information, and trainings); (3) the severity of cancer registration challenges; and (4) the cancer registration timeline, which includes conflict-related events that negatively affected cancer registration.

## DISCUSSION

Cancer registration is essential for NCCPs, efficient resource utilization, screening programs, and rational cancer budget allocations. In that context, this study sought to investigate and map out the present capacities and outputs of cancer registration in the MENAT region and determine thematic barriers facing implementation and utilization of cancer registry data.

Countries with high human development index have more opportunities to develop better cancer surveillance.^1^ Our study highlights that GCC countries, including Bahrain, Kuwait, Oman, Qatar, Saudi Arabia, and the United Arab Emirates, have well-developed PBCRs and faced minimal challenges (Data Supplement). Given the increasing number of expatriates in the Gulf Cooperation Council countries, follow-up on non-national patients with cancer is of particular importance, especially because many travel back to their home countries for treatments and follow-up.^17^ We found in the survey that most countries in the Gulf Cooperation Council do collect data on non-national patients with cancer, especially expatriates. Also, many nationals, eg, Kuwait, might be registered as incident cases and indeed linked to death registration (vital statistics), but they travel to different countries for treatment. Thus, the registration data describe outcomes that are the composite of new cancer therapeutic geographies, rather than NCCP. This is hugely important and very misunderstood and should come out here in a distinct chapter. This is nonforced migration interfaced with forced migration.

On the other hand, armed conflicts and migrations have affected and sometimes halted the process of cancer registration in the region.^1^ Indeed, our study highlights that in areas of protracted conflict, including Yemen and Iraq, cancer registration was directly affected by conflict-related events. These protracted conflicts result in internal displacement and migration to neighboring countries, which further stresses already-weak surveillance systems in the region. It is important to develop mechanisms of surveillance to both displaced and host populations. Of note, the two cancer registries from Turkey, a country that harbors the highest number of refugees in the MENAT region, do not collect information on non-national patients with cancer.

This study is not without limitations. First, not all registries in the MENAT were included in the survey. Second, some registries from the same country reported different answers regarding the number of registries in the country and registration status. All answers were included in the overall analysis of cancer registration, which might skew the data.

The MENAT region has been plagued with recurrent armed conflicts and the associated displacement of millions of people. The long-lasting conflicts and instabilities in Yemen, Syria, Libya, and the Gaza Strip, the transitional government and the rising tensions in Sudan, and the refugee crises in Lebanon, Jordan, and Turkey have repercussions on cancer control and care.^12,13^ Populations in these areas with cancer see their treatment interrupted or stopped, often present with more complications and advanced disease stage, and are rarely enrolled in screening programs. Conflict-affected populations including refugees and forced migrants constitute a unique subpopulation with specific circumstances and needs. Hosting countries should keep their national registries updated with non-national data, including refugee populations, to achieve better surveillance and forecasting capability, which is essential to inform national policy, needs for international assistance, and evidence-based decision making.^18^ This study provides a much-needed understanding of the current landscape and contextual challenges affecting cancer registration in the MENAT.

## Data Availability

The data that support the findings of this study are available from the corresponding author upon reasonable request.

## APPENDIX. ICRIM

Mokhtar Hamdi-Charif, Setif Cancer Registry, Algiers

Eman Hasan Talib Janahi, Bahrain Cancer Registry, Bahrain

Ramez Bedwani, Egypt National Population-Based Cancer Registry, Egypt

Khitam Mohseen, Ministry of Health, Iraqi Cancer Board, Iraqi Cancer Registration Section, Iraq

Omar Nimri, Jordan Cancer Registry, Jordan

Amal Al-Omari, The King Hussein Cancer Center Tumor Registry, Jordan

Amany Elbasmy, Kuwait Cancer Registry, Kuwait

Nada Ghosn, Lebanon National Cancer Registry, Lebanon

Mufid El Mistiri, Benghazi Cancer Registry, Libya

Karima Bendahou, Casablanca Cancer Registry, Morocco

Najwa Al-Lawati, Oman National Cancer Registry, Oman

Huda Lahham, The Palestinian National Cancer Registry, Palestine

Amid Abu Hmaidan, Qatar National Cancer Registry and National Center for Cancer Care and Research Registry, Qatar

Mohammad Al-Shehri, Saudi Cancer Registry, Saudi Arabia

Ahmed Hashim, Sudan National Cancer Registry, Sudan

Feras Al Jerf, Syrian National Cancer Registry, Syria

Mohamed Hsairi, North Tunisian Cancer Registry, Tunisia

Tezer Kutluk, Turkish Pediatric Cancer Registry, Turkey

Sultan Eser, İzmir Cancer Registry, Turkey

Nart Shabsough, UAE Sub-National Cancer Registry, United Arab Emirates

Huda Basaleem, Aden Cancer Registry and Research Center, Yemen

Abdullah Al Nemi, Sana'a Subnational Cancer Registry, Yemen
